# Metagenomic analysis of microbial community dynamics in konjac rhizosphere during soft rot disease progression

**DOI:** 10.1007/s00253-025-13600-4

**Published:** 2025-10-03

**Authors:** Jinping Wu, Jie Zhou, Qinghua Zhao, Chaozhu Yang, Yafan Bai

**Affiliations:** 1 Industrial Crops Institute, Key Laboratory of Vegetable Ecological Cultivation On Highland, Ministry of Agriculture and Rural Affairs, Hubei Key Laboratory of Vegetable Germplasm Enhancement and Genetic Improvement, Hubei Academy of Agricultural Sciences, Wuhan, 430064 Hubei China; 2 Institute of Konjac, Enshi Academy of Agricultural Sciences, Enshi, 445000 Hubei China; 3 College of Horticulture and Forestry Sciences of Huazhong Agricultural University, Wuhan, 430072 Hubei China

**Keywords:** *Amorphophallus konjac*, Rhizosphere microbiome, Soft rot disease, Metagenomics, Biological control

## Abstract

**Abstract:**

*Amorphophallus konjac*, the sole glucomannan-rich species in the Araceae family, faces significant yield and quality losses due to soft rot disease. Understanding the relationship between soil microbial communities and soft rot incidence is critical for sustainable konjac production. Metagenomic profiling was employed to systematically characterize the spatiotemporal dynamics of rhizosphere microbiomes during disease progression. Microbial alpha diversity (Chao1 index) exhibited a significant peak in the rhizosphere of diseased plants at the mature stage, contrasting with stable diversity patterns in healthy and latently infected groups, indicating dysbiosis-associated richness inflation during disease progression. Principal coordinate analysis (PCoA) revealed significant divergence in rhizosphere microbial structures between diseased and healthy/latently infected groups, with higher compositional variability observed in diseased samples. At the phylum level, Chloroflexi and Acidobacteria abundances in healthy mature plants exceeded those in diseased plants by 11.54% and 4.6%, respectively, while pathogenic *Rhizopus arrhizus* and *Rhizopus microsporus* were significantly enriched in diseased mature plants. Correlation analyses demonstrated predominantly negative associations between bacterial species and soil factors, contrasting with positive fungal correlations. KEGG pathway annotation identified carbohydrate metabolism and amino acid synthesis as core microbial functions in the konjac rhizosphere. Collectively, Chloroflexi and Acidobacteria were validated as putative biocontrol agents, while *Rhizopus* spp. emerged as key drivers of soft rot development. These findings provide mechanistic insights for designing microbiome-based biocontrol strategies to mitigate konjac soft rot, offering a sustainable alternative to conventional agrochemical reliance.

**Key points:**

• *Diseased konjac microbial richness peaks; healthy plants enrich Chloroflexi/Acidobacteria.*

• *Rhizopus pathogens drive soft rot; bacteria and fungi show opposing soil factor links.*

• *Lays groundwork for microbiome approaches to cut agrochemicals in konjac rot control.*

**Supplementary Information:**

The online version contains supplementary material available at 10.1007/s00253-025-13600-4.

## Introduction

*Amorphophallus konjac*, a distinct glucomannan-rich species within the Araceae family, has garnered substantial scientific and industrial interest owing to the unique gelation and film-forming properties of konjac glucomannan, driving its diverse applications in food technology, biomedicine, and material science (Devaraj et al. 2019; Tan et al. 2021). Despite its economic significance, sustainable cultivation of *A. konjac* is severely challenged by high susceptibility to soft rot disease, primarily caused by *Pectobacterium* spp. This soil-borne pathogen induces rapid maceration of corms and petioles, with infection rates typically ranging from 30–50% and exceeding 80% in severe outbreaks, often leading to complete yield loss (He et al. 2022). Current management strategies, including chemical pesticides and disease-resistant cultivars, are largely ineffective due to the pathogen’s persistent soil survival and complex host-microbiome interactions.

The rhizosphere microbiome serves as a critical determinant of plant-pathogen dynamics, acting as the first line of defense against soil-borne pathogens (Solomon et al. 2024). In diverse crop systems, pathogens must overcome resident microbial communities to establish infections, as demonstrated in tomato bacterial wilt (Wei et al. 2019), tobacco root rot (Gao et al. 2019), and banana Fusarium wilt (Shen et al. 2015). Disease progression systematically reshapes microbial community composition; for example, increased Deinococcus-Thermus and Firmicutes abundances in cotton verticillium wilt (Zhang et al. 2010) and reduced Proteobacteria populations in citrus Huanglongbing (Trivedi et al. 2011) highlight pathobiome-specific signatures. In *A. konjac*, prior studies (Wu et al. 2017) have only preliminarily characterized rhizosphere microbial diversity but have not resolved temporal assembly patterns during latent infection, functional shifts of the microbiome across disease stages, or early predictive biomarkers of disease onset.

Here, we hypothesize that the dichotomous disease outcomes (healthy vs. severely infected) in *A. konjac* under uniform environmental conditions originate from early divergences in rhizosphere microbiome assembly, consistent with emerging evidence of microbiome-mediated disease suppression in other plant systems (Gu et al. 2022). To test this, we employ a novel “rootbox” system for non-destructive, stage-specific sampling of rhizosphere soil from individual plants (Wei et al. 2019), combined with a retrospective tracking strategy, to dynamically correlate microbiome succession with plant health status at the single-plant resolution. This approach aims to uncover mechanistic links between microbial community dynamics and disease progression, providing a foundation for developing innovative, microbiome-based biocontrol strategies to support sustainable *A. konjac* production.

## Materials and methods

### Rhizosphere soil collection system and sampling design

Experimental trials were conducted at the Agricultural High Technology Zone (30.3172°N, 109.4772°E; 421.5 m a.s.l.) in Enshi Tujia and Miao Autonomous Prefecture, Hubei Province, China. A specialized rootbox system was employed to non-destructively collect rhizosphere soil from individual *A.konjac* plants across three developmental stages (initial, vegetative, maturity). The system comprised nine detachable nylon mesh compartments (dimensions: height = 136 mm, width = 18–21 mm, thickness = 1–2 mm; pore size = 150 μm), enabling stage-specific sampling without plant disturbance. Soil samples were passed through a 2-mm sieve to remove plant debris, homogenized by gentle vortexing, and aliquoted (4 g per tube) to minimize microbial biomass variation. The rootbox soil originated from a 5-year continuous cropping plot with severe soft rot disease history, ensuring natural pathogen pressure.

### Field experimental design and retrospective sampling strategy

Tissue-cultured seedlings of *Amorphophallus konjac* K. Koch cv. Qingjiang were obtained from the Konjac Research Institute (Enshi, China) and cultivated under uniform greenhouse conditions (consistent soil properties, temperature, humidity, and light cycles). On April 10, 2023, 200 seedlings were transplanted into pre-assembled rootboxes and subjected to standardized agronomic management. Rootboxes were randomly positioned to minimize spatial variability, ensuring microbiome differences reflected biological treatments rather than environmental heterogeneity.

Disease progression was monitored at three phenological stages:

- Initial stage (July 10): 25.0% infection rate
- Vegetative stage (August 10): 46.0% infection rate
- Maturity stage (September 10): 65.0% infection rate

At each stage, three nylon compartments were randomly harvested per rootbox. Samples from the same plant across stages were pooled, homogenized, and stored at − 80 ℃. At maturity, plants were classified into three health categories via quantitative PCR (qPCR) of stem tissue:

- Healthy: No symptoms + pathogen load < 10^2^ CFU/g
- Latently infected: Asymptomatic + 10^2^–10^6^ CFU/g
- Diseased: Symptomatic + pathogen load > 10^6^ CFU/g

Soil samples were retrospectively labeled based on final plant health:

- Diseased-associated: DIS (initial), DVS (vegetative), DMS (maturity)
- Latently infected-associated: LIS, LVS, LMS
- Healthy-associated: HIS, HVS, HMS

For metagenomic analysis, six biological replicates per health category × 3 stages yielded 54 samples (18 samples/stage).

### Soil physicochemical analysis

Physicochemical analysis was conducted to assess the soil’s physicochemical characteristics under different plant health statuses and growth stages. Triplicate measurements were carried out to evaluate the field soil conditions. Soil pH was measured by preparing a mixture of soil and CO_2_-free deionized water at a 1:2.5 (w/v) ratio. Organic matter (OM), available nitrogen (AN), available phosphorus (AP), and available potassium (AK) were determined following established protocols (Wu et al. 2020).

### DNA extraction, qPCR, and metagenomic sequencing

Soil DNA was extracted using the Mag-Bind® Soil DNA Kit (Omega Bio-tek), and plant tissue DNA via the Plant Genomic DNA Kit (Tiangen). DNA concentration/purity was assessed by TBS-380 and NanoDrop 2000. For qPCR, 2 μL DNA templates were analyzed in 20 μL reactions (2 × SYBR Green Master Mix, 0.4 μM primers/probe) using thermal cycling: 95 ℃ × 5 min, followed by 40 cycles of 95 ℃ × 15 s and 60 ℃ × 30 s. Pathogen abundance was calculated using serial dilution standard curves (Wu et al. 2011).

Metagenomic libraries were prepared by fragmenting DNA to − 400 bp (Covaris M 220) and using NEXTFLEX Rapid DNA-Seq. Sequencing was performed on an Illumina NovaSeq 6000 platform. Data were analyzed on the Majorbio Cloud Platform:

- Quality assessment: FastQC
- Processing: Trimmomatic (adapter removal), IDBA-UD (assembly), Prodigal (ORF prediction), CD-HIT (redundancy removal)
- Taxonomic annotation: DIAMOND v2.0.15 (*E*-value ≤ 1e − 5) against NCBI NR, with LCA assignment via MEGAN6 v6.20.6
- Functional profiling: KEGG pathway annotation (GhostKOALA server, level 3)

### Microbiome profiling and data processing

Raw sequencing data were subjected to stringent quality control using fastp v0.20.0 to remove adapters, low-quality reads (length < 50 bp, Phred score < 20), and sequences containing ambiguous bases (N). High-quality non-host reads were de novo assembled into contigs (≥ 300 bp) using MEGAHIT v1.1.2 with default parameters (k-mer range: 21–141, step size: 10), retaining only contigs with coverage ≥ 2 × to ensure assembly reliability.

ORFs were predicted with Prodigal v2.6.3, and redundant sequences clustered via CD-HIT v4.8.1 (95% identity, 90% coverage). Taxonomic annotation used DIAMOND v2.0.15 against NCBI NR (*E*-value ≤ 1e − 5, bit-score ≥ 50, coverage ≥ 60%); functional profiling employed GhostKOALA for KEGG pathway mapping (level 3).

Data were normalized via TMM (edgeR). Alpha diversity (Chao1, Shannon, Simpson) was computed in R (vegan), with group differences tested by Kruskal–Wallis *H* tests (Dunn’s post hoc, FDR < 0.05). Beta diversity was visualized via PCoA (Bray–Curtis distances), with PERMANOVA (999 permutations, adonis) for group comparisons. Differential abundance of taxa and KEGG pathways was identified using DESeq2 (FDR < 0.05). Spearman correlations between microbes/functions and soil factors (*p* < 0.05) were visualized as heatmaps (pheatmap). All analyses used R v4.2.1, with visualizations (PCoA, bar plots, Venn diagrams) generated via ggplot2 and VennDiagram.

## Results

### Disease status classification in konjac plants

Through systematic evaluation of symptomatic manifestations and quantitative PCR (qPCR) detection of *Pectobacterium chrysanthemi* in stem tissues, the experimental population (*n* = 200) exhibited distinct health stratification. Additionally, qPCR analysis of rhizosphere soil samples revealed a significant positive correlation between soil-borne pathogen abundance and plant disease severity (data not shown), consistent with the stem tissue detection results. Among them, 65.0% (*n* = 130) were classified as diseased, showing characteristic soft rot symptoms and pathogen loads exceeding 10^6^ CFU/g. Meanwhile, 21.5% (*n* = 43) were latently infected, remaining asymptomatic yet harboring pathogens at levels ranging from 10^2^ to 10^6^ CFU/g. Additionally, 13.5% (*n* = 27) were considered healthy, displaying neither symptoms nor detectable pathogens (< 10^2^ CFU/g). This categorical distribution demonstrated significant association between symptom development and pathogen proliferation (*χ*^2^ = 167.3, df = 2, *p* < 0.0001), confirming the biological relevance of the classification criteria.

### Taxonomic composition based on NR database annotation

Gene sequences of microorganisms in konjac rhizosphere soil were annotated by species through comparison with the NR database (Table 1). As shown in the table, bacteria were the predominant microorganisms in each sample, accounting for 99.04% of the total species, followed by Heunggongvirae (0.48%) and Archaea (0.18%), while fungi were relatively rare, comprising only 0.09%. In this study, bacteria and fungi with potential functional significance were selected for subsequent analysis.

**Table 1:** The taxonomic distribution of microbial communities in konjac rhizosphere soil

Domain	Kingdom/phylum	Read counts	Relative abundance (%)
Bacteria	All phyla	431,501,482	99.04
Viruses	Heunggongvirae	2,080,076	0.48
	Bamfordvirae	163,354	0.04
	Other viral taxa	91,796	0.02
Archaea	All phyla	795,568	0.18
Eukaryota	Viridiplantae	436,704	0.10
	Fungi	392,790	0.09
	Metazoa	115,064	0.03
Unclassified	-	85,498	0.02

Data filtered at ≥ 0.01% relative abundance threshold. Viral subcategories consolidated under “Viruses” domain for hierarchical clarity

### Alpha diversity index of microbial communities in rhizosphere of konjac

The α-diversity indices of bacterial and fungal communities in the konjac rhizosphere, across different health statuses and growth stages, are presented in Tables 2 and 3. For both healthy and diseased plants, the Chao1 indices (reflecting community richness) for bacteria and fungi were higher during the mature stage compared to the initial stage, while the Simpson indices (reflecting community diversity) decreased correspondingly. The highest Chao1 values and the lowest Simpson indices were observed in the diseased plants at the mature stage (DMS). These results suggest that microbial community richness and diversity in the konjac rhizosphere vary with growth stage and health status, peaking during the mature stage of diseased plants.

**Table 2:** The alpha diversity indices of bacterial communities in the rhizosphere of konjac under different health statuses and growth stages

Sample	Chao1	Shannon	Simpson
DIS	366.67 ± 10.975 ab	4.0990 ± 0.0126 a	0.0572 ± 0.0013 b
DVS	372.33 ± 51.541 ab	4.1972 ± 0.3863 a	0.0599 ± 0.0261 b
DMS	413.00 ± 6.429 a	2.3849 ± 0.1781 b	0.2996 ± 0.0549 a
HIS	348.33 ± 12.811 ab	4.2582 ± 0.1576 a	0.0590 ± 0.0105 b
HVS	338.33 ± 11.893 b	3.8288 ± 0.0262 a	0.0855 ± 0.0017 b
HMS	351.67 ± 20.078 ab	4.1484 ± 0.0097 a	0.0685 ± 0.0060 b
LIS	325.33 ± 12.441 b	3.9642 ± 0.0585 a	0.0682 ± 0.0061 b
LVS	397.33 ± 10.682 ab	4.1046 ± 0.0036 a	0.0607 ± 0.0015 b
LMS	374.33 ± 20.019 ab	3.9380 ± 0.1047 a	0.0699 ± 0.0075 b

(1) Different lowercase letters indicate significant differences between treatments (*p* < 0.05). (2) Abbreviations: *DIS*, diseased plants at initial stage; *DVS*, diseased plants at vegetative stage; *DMS*, diseased plants at mature stage; *HIS*, healthy plants at initial stage; *HVS*, healthy plants at vegetative stage; *HMS*, healthy plants at mature stage; *LIS*, latently infected plants at initial stage; *LVS*, latently infected plants at vegetative stage; *LMS*, latently infected plants at mature stage

**Table 3:** The alpha diversity indices of fungal communities in the rhizosphere of konjac under different health statuses and growth stages

Sample	Chao1	Shannon	Simpson
DIS	21,340.67 ± 147.931 d	5.3896 ± 0.0633 b	0.0228 ± 0.0012 b
DVS	21,417.33 ± 189.711 d	5.4278 ± 0.1716 b	0.0252 ± 0.0036 ab
DMS	22,876.33 ± 39.048 a	6.0730 ± 0.0091 a	0.0109 ± 0.0002 c
HIS	20,792.67 ± 223.570 e	5.2049 ± 0.1631 b	0.0317 ± 0.0046 a
HVS	21,437.00 ± 144.133 d	5.1836 ± 0.0556 b	0.0313 ± 0.0020 a
HMS	22,436.33 ± 185.869 ab	5.3875 ± 0.0557 b	0.0259 ± 0.0012 ab
LIS	20,652.33 ± 298.241 e	5.3089 ± 0.1003 b	0.0267 ± 0.0025 ab
LVS	21,810.33 ± 104.260 cd	5.2339 ± 0.0356 b	0.0293 ± 0.0011 ab
LMS	22,183.00 ± 125.012 bc	5.2924 ± 0.0281 b	0.0279 ± 0.0010 ab

(1) Different lowercase letters indicate significant differences between treatments (*p* < 0.05). (2) Abbreviations: *DIS*, diseased plants at initial stage; *DVS*, diseased plants at vegetative stage; *DMS*, diseased plants at mature stage; *HIS*, healthy plants at initial stage; *HVS*, healthy plants at vegetative stage; *HMS*, healthy plants at mature stage; *LIS*, latently infected plants at initial stage; *LVS*, latently infected plants at vegetative stage; *LMS*, latently infected plants at mature stage

**Fig. 1:**
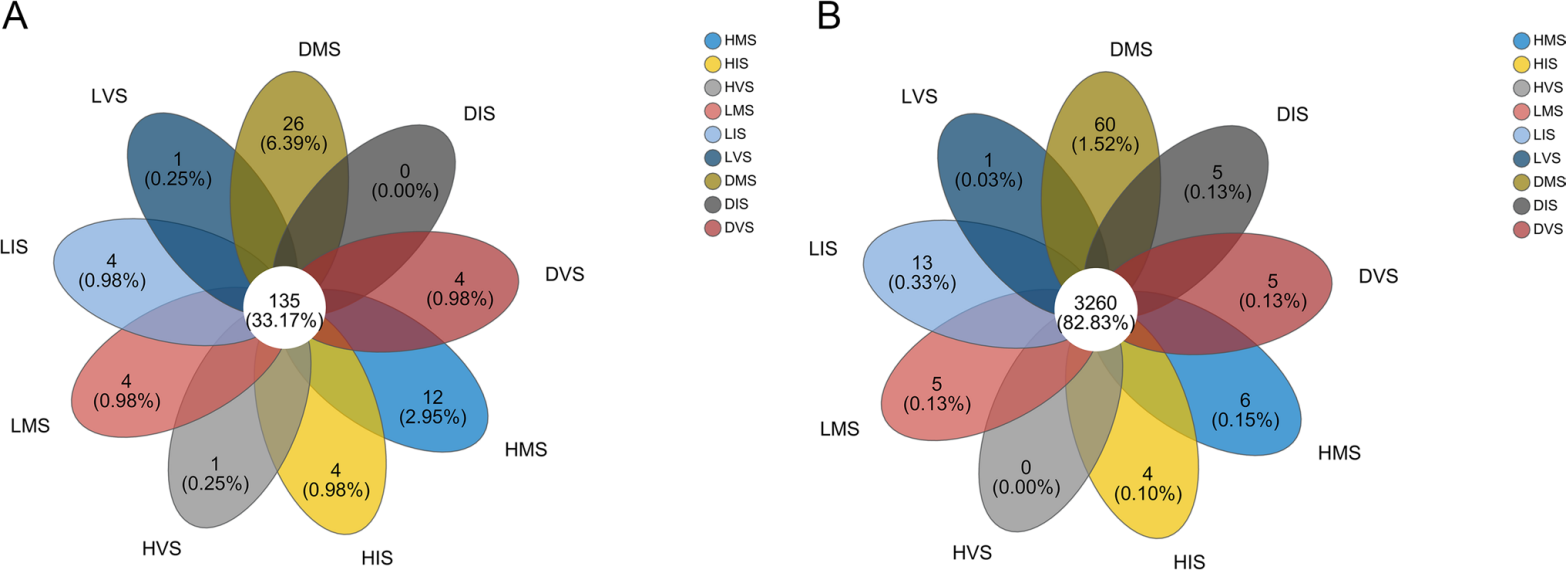
The Venn diagram of fungal (**A**) and bacterial (**B**) communities in the rhizosphere of konjac under different health statuses and growth stages

The similarity in bacterial and fungal species composition across health statuses and growth stages was visualized using Venn diagrams (Fig. 1). Notably, 6.39% of fungal species and 1.52% of bacterial species were unique to the diseased mature stage (DMS), compared to 2.95% and 0.15%, respectively, in the healthy mature stage (HMS). This highlights a greater abundance of unique rhizosphere microbial species during the mature stage of konjac soft rot disease (DMS).

### Beta diversity of microbial communities in the rhizosphere of konjac

**Fig. 2:**
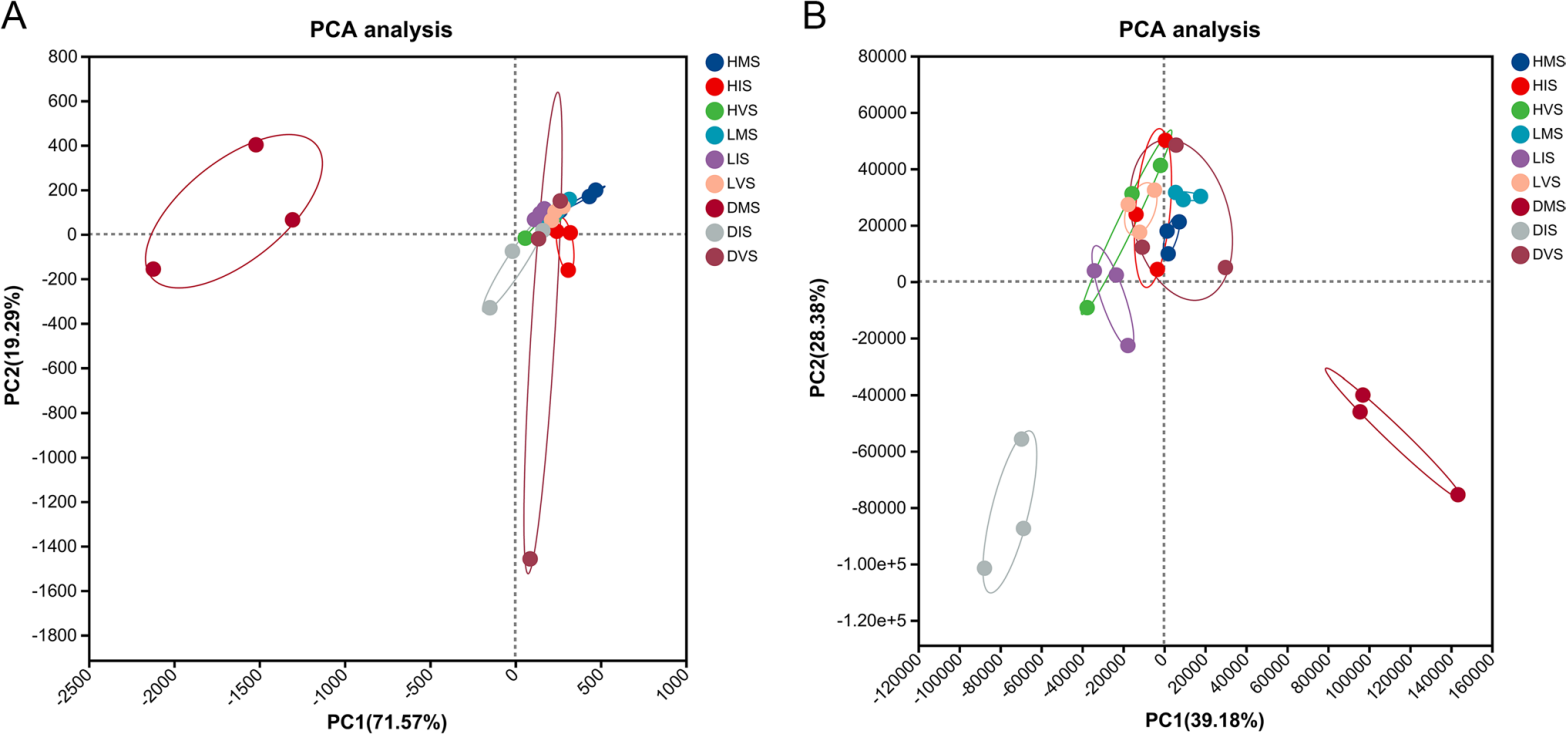
The principal component analysis (PCA) of fungal (**A**) and bacterial (**B**) communities in the rhizosphere of konjac under different health statuses and growth stages

Principal component analysis (PCA) was used to evaluate differences and similarities in bacterial and fungal communities within the konjac rhizosphere across varying health statuses and growth stages (Fig. 2). The results showed significant community structuring along PC1 (39.18% variance for bacteria; 71.57% for fungi), with PERMANOVA confirming health status as the primary driver (bacteria: *F* = 12.6, *p* < 0.001; fungi: *F* = 18.3, *p* < 0.001). Diseased samples exhibited higher dispersion (Bray–Curtis distances: 0.58 ± 0.12), indicative of increased community instability.

Variations related to growth stages were primarily distributed along PC2 (explaining 19.29% and 28.38% of variance for bacteria and fungi, respectively), indicating that health status was the dominant factor shaping bacterial and fungal community structure in the konjac rhizosphere, while growth stage played a secondary role. Furthermore, diseased sample points exhibited greater dispersion compared to healthy and latently infected samples, suggesting higher variability in microbial diversity within the rhizosphere soil of konjac plants affected by soft rot disease.

### Composition of microbial communities in the rhizosphere of konjac

**Fig. 3:**
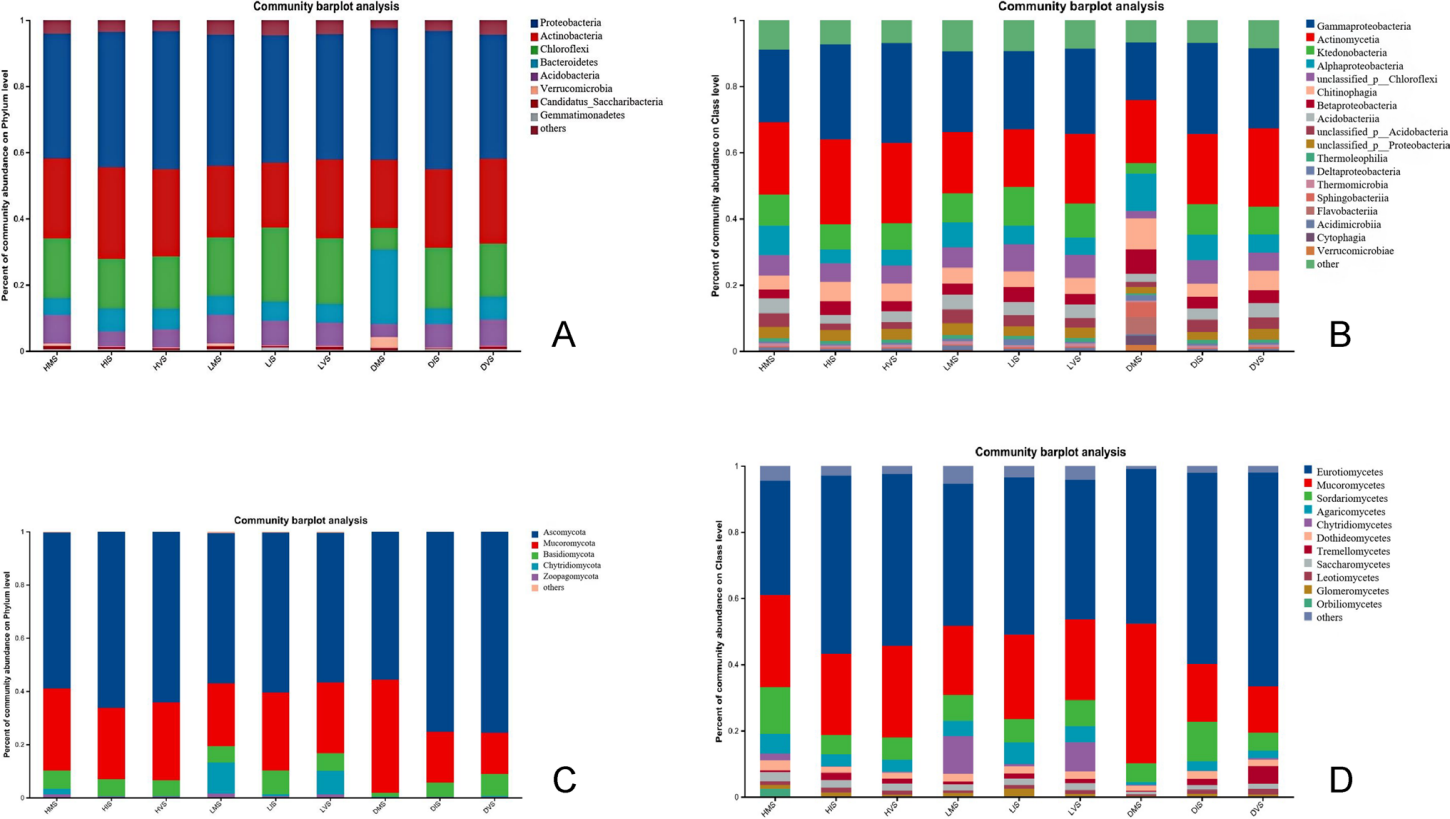
The relative abundance of dominant bacterial (**A** phylum, **B** class) and fungal (**C** phylum, **D** class) taxa in the konjac rhizosphere under different health statuses and growth stages

Across all samples, bacterial communities were annotated into 160 phyla, 268 classes, 478 orders, 989 families, 3936 genera, and 29,372 species, while fungal communities comprised nine phyla, 36 classes, 99 orders, 250 families, 407 genera, and 828 species. The relative abundances of dominant bacterial and fungal taxa at each taxonomic level were analyzed (Fig. 3).

The dominant bacterial phyla in the konjac rhizosphere were Proteobacteria and Actinobacteria, collectively accounting for 62.25% of the community, followed by Chloroflexi, Bacteroidetes, Acidobacteria, Verrucomicrobia, Candidatus_Saccharibacteria, and Gemmatimonadetes. Both growth stage and health status influenced the relative abundance of dominant taxa. In diseased plants at the mature stage, the abundance of Chloroflexi (6.55%) and Acidobacteria (4.03%) was notably lower compared to healthy plants at the same stage (18.09% and 8.69%, respectively; Fig. 3A). Conversely, Bacteroidetes (22.35%) and Verrucomicrobia (3.27%) were enriched in diseased plants relative to healthy plants (5.05% and 0.63%, respectively), suggesting their potential role in disease response (Fig. 3A). At the class level, dominant taxa such as Ktedonobacteria, unclassified Chloroflexi, unclassified Acidobacteria, and unclassified *Proteobacteria* were reduced in diseased mature plants. In contrast, Alphaproteobacteria, Chitinophagia, Betaproteobacteria, Sphingobacteriia, Flavobacteriia, Cytophagia, and Verrucomicrobiae were more abundant in diseased plants (Fig. 3B).

Dominant fungal phyla included Ascomycota, Mucoromycota, and Basidiomycota. At the mature stage, diseased konjac exhibited lower abundances of Chytridiomycota and Zoopagomycota compared to healthy and latently infected plants (Fig. 3C). During the initial growth stage, fungal abundances across health states were similar. In the vegetative stage, latently infected plants showed higher Chytridiomycetes abundance than healthy or diseased plants. By the mature stage, Chytridiomycetes abundance in diseased plants was significantly lower than in healthy and latently infected plants. Healthy plants displayed higher Chytridiomycetes abundance at maturity compared to earlier stages, while latently infected plants showed increased Chytridiomycetes abundance at later stages. Disease plant exhibited elevated Mucoromycota abundance at the mature stage relative to earlier stages (Fig. 3D).

**Fig. 4:**
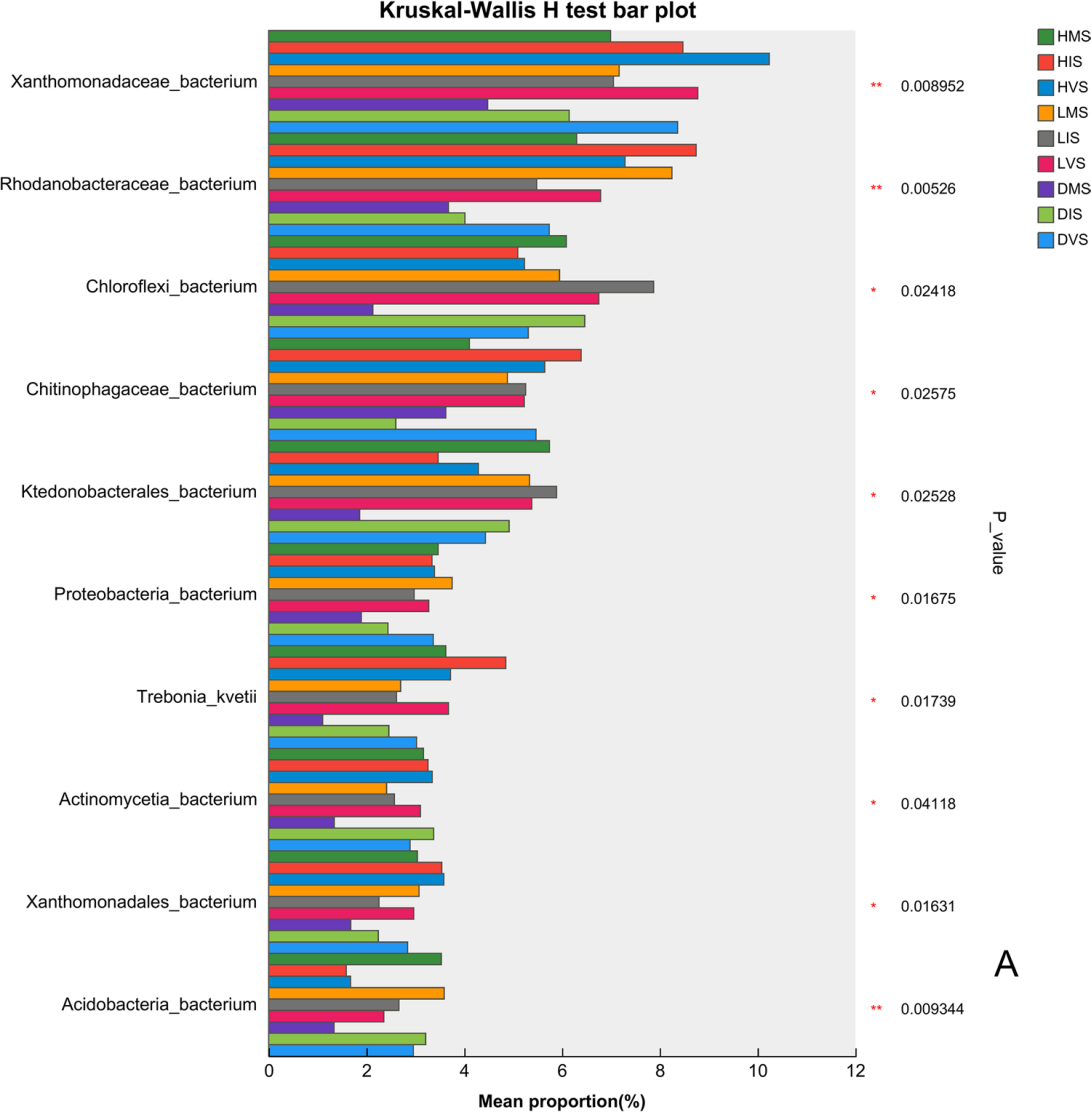

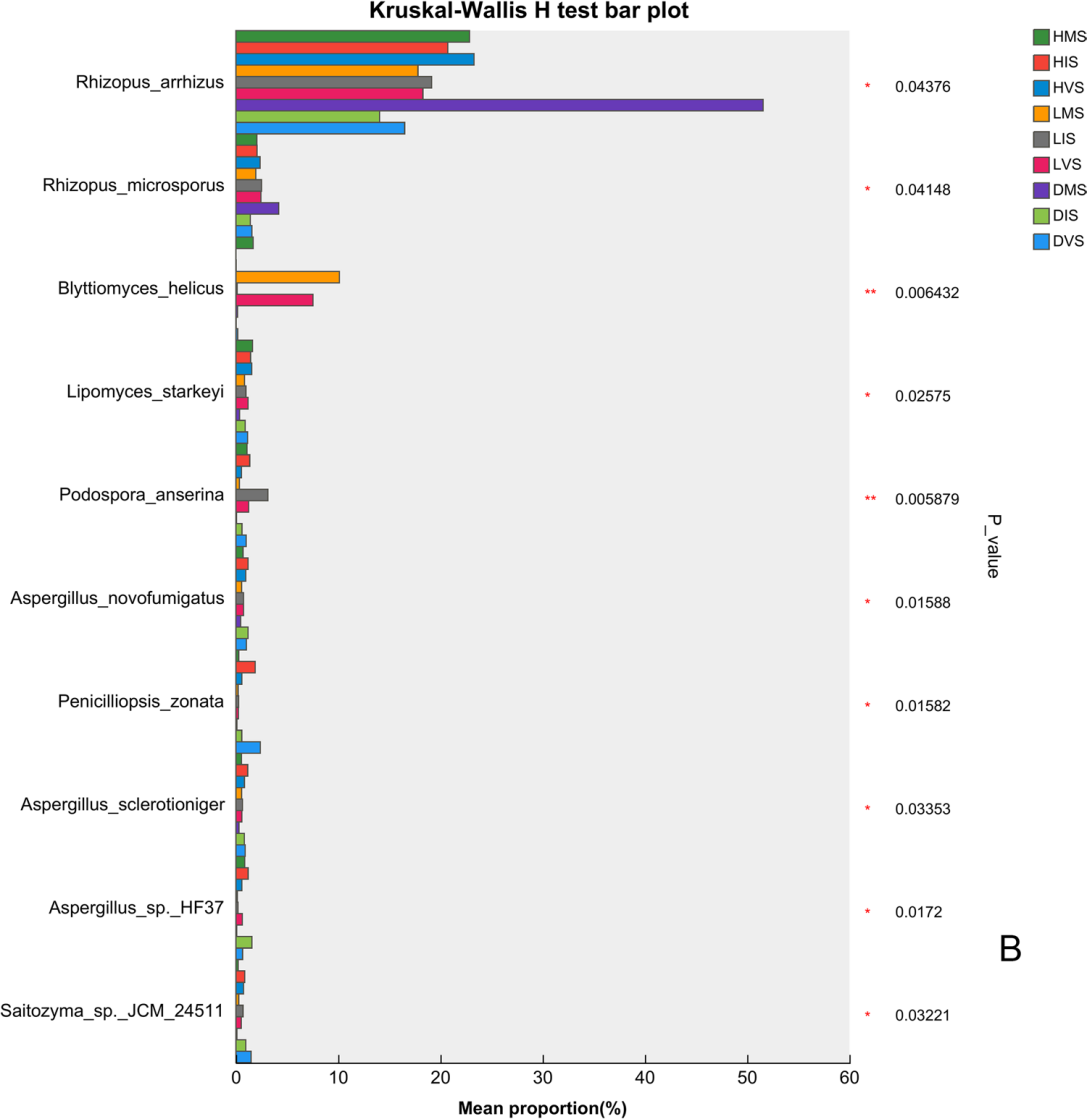
The differential species analysis of **A** bacterial and **B** fungal communities in the konjac rhizosphere

The Kruskal–Wallis *H* test identified significant differences in the abundance of bacterial and fungal taxa across varying health conditions and growth stages (Fig. 4). Among the top 10 bacterial taxa, *Xanthomonadaceae_bacterium* (family level), *Rhodanobacteraceae_bacterium* (family level), and *Acidobacteria_bacterium* (phylum level) showed highly significant differences, with their abundances lowest under disease conditions. *Xanthomonadaceae_bacterium* peaked during the vegetative stage, following the trend: healthy > latently infected > diseased (Fig. 4A). For fungi, the species *Rhizopus arrhizus* and *Rhizopus microsporus* were significantly enriched in diseased mature plants, whereas the species *Blyttiomyces helicus* was abundant in latently infected mature plants (Fig. 4B).

### Microbial-enzyme gene associations revealed by Spearman correlation analysis

**Fig. 5:**
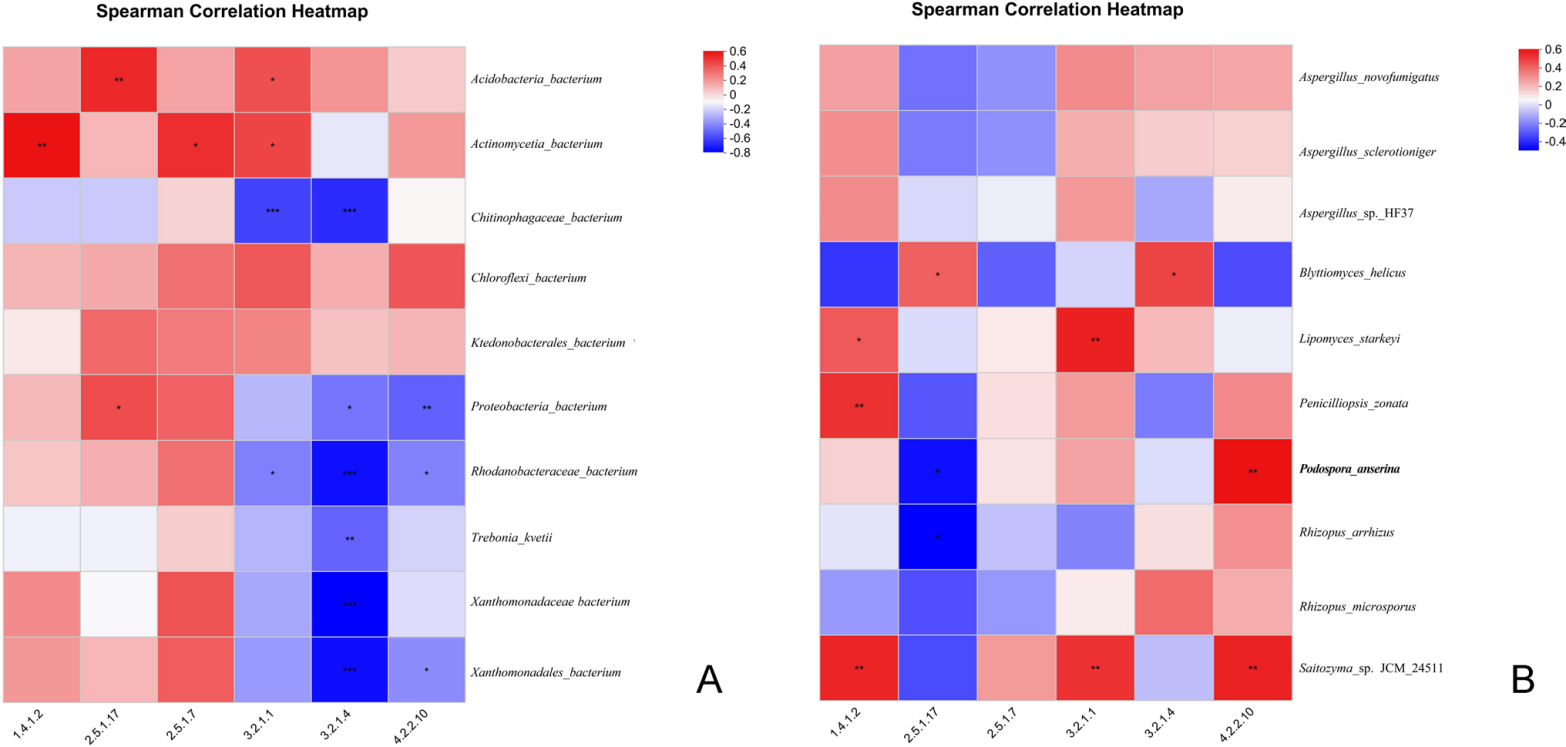
The Spearman correlation heatmap between enzyme Gene and microbial species **A** bacterial and **B** fungal

To decipher the associations between key enzyme-encoding genes and dominant microbial taxa in the soil, Spearman’s rank correlation analysis was performed using the pheatmap package in R (v4.2.1), with significance determined by Benjamini–Hochberg false discovery rate (FDR) correction (*α* < 0.05). Enzyme-encoding genes were identified through KEGG pathway annotation (level 3) via GhostKOALA, focusing on cell wall-degrading enzymes (e.g., cellulase, pectate lyase) with EC numbers (EC 3.2.1.4, EC 4.2.2.10) that are functionally linked to plant tissue maceration. Gene abundances were normalized using TMM (edgeR) to ensure comparability across samples, and results were visualized in heatmaps (Fig. 5).

For bacterial taxa, distinct correlation patterns were observed: *Xanthomonadaceae_bacterium* (family level) and *Rhodanobacteraceae_bacterium* (family level) exhibited significant negative correlations with the cellulase-encoding gene EC 3.2.1.4 (Fig. 5A), suggesting that these bacteria may participate in soil carbon cycle regulation by inhibiting cellulolytic enzyme activity.

For fungal taxa (Fig. 5B), the species *Rhizopus arrhizus* and *Rhizopus microsporus* showed significant positive correlations with both the pectate lyase-encoding gene EC 4.2.2.10 and cellulase-encoding gene EC 3.2.1.4, indicating their potential role in modulating cell wall-degrading enzyme activities to facilitate host tissue maceration during konjac soft rot progression. Notably, the species *Podospora anserina* displayed a positive correlation with EC 4.2.2.10 but negative correlations with other enzyme-encoding genes, reflecting the complex metabolic interactions between fungal taxa and enzyme systems in the soil.

### Correlations between rhizosphere microbial taxa and soil physicochemical properties

**Fig. 6:**
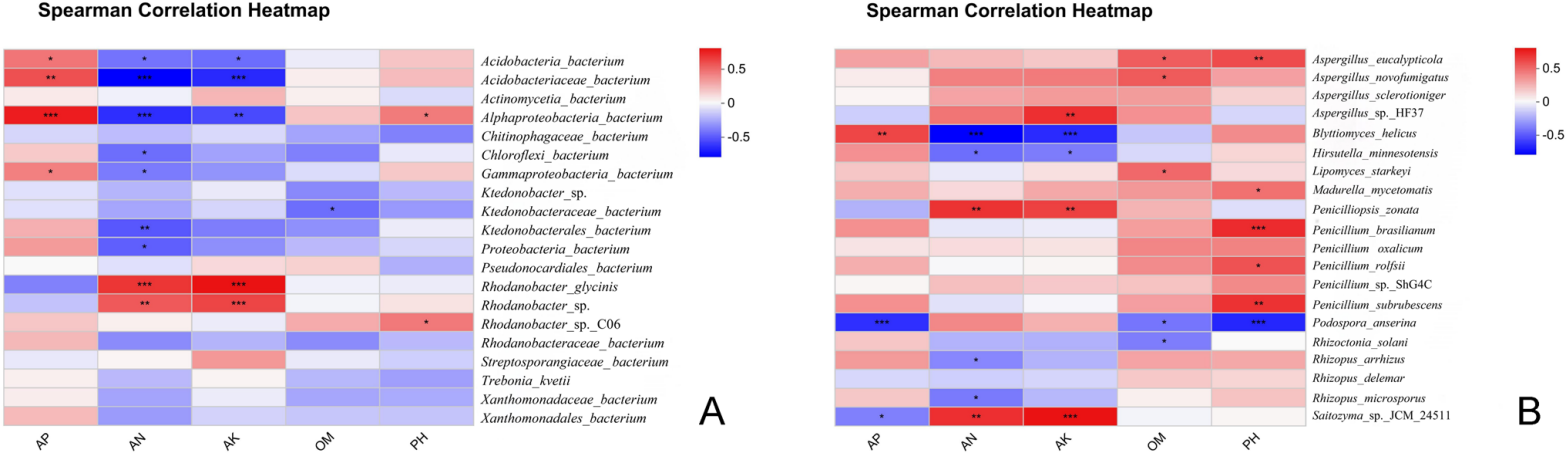
The Spearman correlation heatmap between soil factors and microbial species **A** bacterial and **B** fungal. (Notes: OM, organic matter; AN, available nitrogen; AP, available phosphorus; AK, available potassium)

To decipher the associative relationships between rhizosphere microbial communities and edaphic properties, Spearman’s rank correlation coefficients were calculated for microbial taxa (at genus/species level) and soil factors including organic matter (OM), available nitrogen (AN), available phosphorus (AP), available potassium (AK), and pH. Detailed analyses of soil physicochemical properties, including variance analysis results, are presented in supplementary Table 1 (see supplementary Table 1 for detailed data). The heatmap (Fig. 6) visualizes Spearman correlation coefficients (*r*) scaled by color intensity, where red denotes positive correlations, blue indicates negative correlations, and color saturation corresponds to the magnitude of |*r*|. Significance was determined using Benjamini–Hochberg false discovery rate (FDR) correction at *α* < 0.05, accounting for multiple testing biases.

Key observations on correlation patterns reveal that most bacterial taxa exhibited significant correlations with soil nutrients and pH, reflecting potential niche specialization (Fig. 6). For instance, *Alphaproteobacteria bacterium* (class level) and *Rhodanobacter* sp. *C06* (species level) showed strong positive correlations with pH (*r* = 0.68 and 0.72, respectively), suggesting alkaliphilic preferences. Conversely, *Ktedonobacteraceae bacterium* (family level) displayed a robust negative correlation with OM (*r* =  − 0.59), possibly indicating reduced fitness in high-OM environments or indirect effects via pathogen-driven organic matter decomposition. *Acidobacteria bacterium* (phylum level) and *Rhodanobacter glycinis* (species level) were positively associated with available nitrogen (AN) and potassium (AK) (*r* = 0.55–0.62), implying roles in nutrient cycling (Fig. 6A).

Fungal taxa showed distinct correlation patterns, with the species *Podospora anserina* and *Rhizopus arrhizus* demonstrating positive links to pH (*r* = 0.51 and 0.48, respectively). The species *Aspergillus novofumigatus* exhibited a notable negative correlation with OM (*r* =  − 0.61), potentially reflecting competition with bacterial decomposers in organic-rich niches. Extreme values included the species *Rhizopus microsporus* with a moderate positive correlation to AP (*r* = 0.45), suggesting phosphate-dependent growth (Fig. 6B).

### KEGG functional profiling of konjac rhizosphere microbiome

**Fig. 7:**
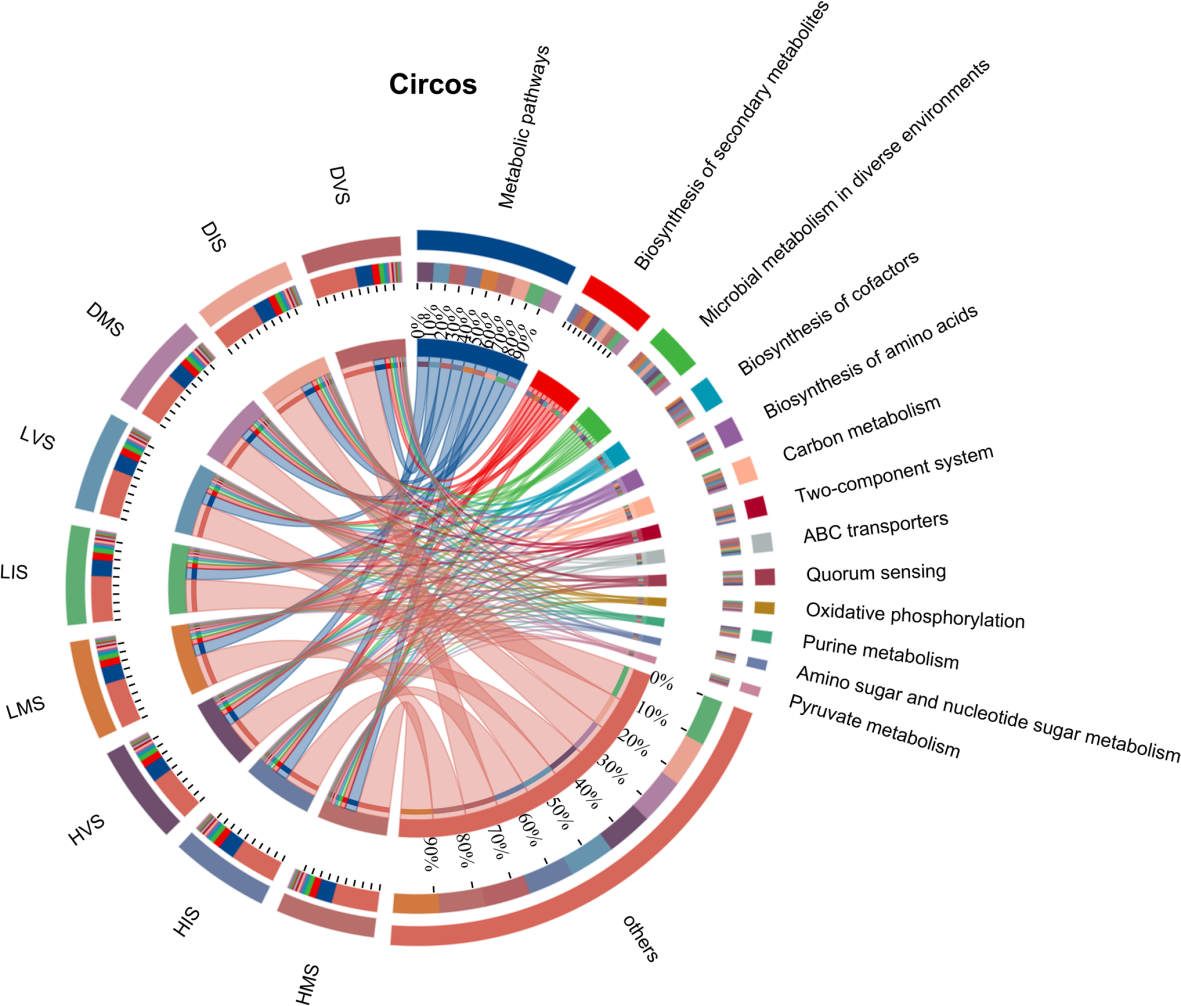
The relative abundance of KEGG-annotated pathways in the konjac rhizosphere under different health statuses and growth stages

To characterize functional dynamics of the rhizosphere microbiome across plant health statuses (healthy, latently infected, diseased) and growth stages (initial, vegetative, mature), metagenomic sequences were annotated against the KEGG database using GhostKOALA (level 3 pathways), with hierarchical functional profiles analyzed at three levels (level 1: primary functional categories; level 2: subcategories; level 3: specific pathways/enzymes). Differential abundance statistics were applied to identify stage- and health-specific functional shifts (Fig. 7).

At level 1, dominant categories included “Metabolism” (42.3% average relative abundance), “Genetic Information Processing” (18.7%), and “Environmental Information Processing” (15.2%). Level 2 subcategories within “Metabolism” were led by “Carbohydrate Metabolism” (17.6%), “Amino Acid Metabolism” (10.3%), and “Biosynthesis of Secondary Metabolites” (7.80%), consistent with the core role of rhizosphere microbiomes in nutrient cycling.

Level 3 pathways (specific metabolic pathways and enzyme-coding genes) revealed the most biologically relevant differences, including “Starch and sucrose metabolism” (EC 3.2.1.26, EC 3.2.1.4) involved in carbohydrate degradation, which showed 2.3-fold higher abundance in healthy mature plants (HMS) compared to diseased mature plants (DMS; *p* < 0.01); “Phenylalanine metabolism” (EC 4.3.1.24, EC 2.6.1.57) linked to plant defense compound synthesis, enriched in HMS (1.8-fold vs. DMS; *p* < 0.05); and “Pectin degradation” (EC 4.2.2.10, pectate lyase) and “Cellulose degradation” (EC 3.2.1.4, cellulase), which were significantly upregulated in DMS (2.1-fold and 1.9-fold vs. HMS, respectively; *p* < 0.01) and aligned with the cell wall-degrading enzyme activities observed in pathogenic *Rhizopus* spp. (Fig. 5B).

Statistical analyses via Kruskal–Wallis *H* tests with Benjamini–Hochberg FDR correction (*α* < 0.05) confirmed these level 3 shifts. For example, “Amino Sugar and Nucleotide Sugar Metabolism” (level 3), critical for glycan synthesis, was reduced by 42% in DMS compared to HMS (*p* < 0.01), indicating impaired carbohydrate metabolism during severe infection. Conversely, “Biosynthesis of Cofactors” (level 3, e.g., EC 2.7.7.2, involved in vitamin B synthesis) was enriched in healthy plants (1.5-fold vs. DMS; *p* < 0.05), potentially reflecting enhanced stress resistance.

Temporal dynamics at level 3 revealed stage-specific trends: “Two-Component Systems” (level 3, EC 2.7.13.3, sensor kinases) peaked in the vegetative stage across all groups, suggesting heightened microbial environmental adaptation during active plant growth. “Oxidative Phosphorylation” (level 3, EC 7.1.1.2, NADH dehydrogenase) was elevated in latently infected plants, indicating altered energy metabolism during subclinical pathogen colonization.

Enzyme-level annotations (level 3) were generated by mapping predicted ORFs (via Prodigal v2.6.3) to KEGG Orthologs (KO) using GhostKOALA, followed by extraction of EC numbers and their corresponding pathways. Normalization of enzyme gene abundances was performed via TMM (edgeR) to ensure cross-sample comparability. These level 3 data were integrated with microbial taxonomic profiles to identify associations (e.g., positive correlations between *Rhizopus* spp. and pectate lyase genes; Fig. 5B), linking specific taxa to functional capacities.

## Discussion

### Changes in the rhizosphere microbial community

The diversity and composition of rhizosphere microbial communities are closely linked to the occurrence of soil-borne diseases (Li et al. 2015). This study revealed that microbial community richness and diversity in the konjac rhizosphere peaked during the mature stage of diseased plants. The Chao1 indices for bacterial and fungal communities in diseased konjac rhizosphere were 413.00 and 22,876.33, respectively, indicating higher microbial diversity in diseased rhizosphere soil compared to healthy plants. This aligns with findings in pepper plants affected by bacterial wilt, where diseased rhizosphere soil exhibited greater microbial diversity than healthy soil (Liu et al. 2021). Principal coordinate analysis (PCoA) demonstrated significant differences in bacterial and fungal community structures across varying plant health conditions and growth stages, with diseased samples showing higher dispersion. Similarly, Zhang et al. (2023) reported distinct rhizosphere microbial community structures between healthy Chinese cabbage and clubroot-diseased plants.

At the phylum level, the relative abundances of Chloroflexi and Acidobacteria in healthy plants at the mature stage were 11.54% and 4.66% higher, respectively, than in diseased plants. These taxa are recognized as key biological indicators of soil disease suppression (Wang et al. 2019; Wang et al. 2020a, b). Acidobacteria actively interact with plants as growth-promoting bacteria (Kielak et al. 2016), while both Chloroflexi and Acidobacteria contribute to konjac’s resistance against soft rot infection.

In diseased plants, the abundance of Chytridiomycota was significantly reduced compared to healthy plants (Liao et al. 2021), whereas Mucoromycota was enriched in soils of root-diseased plants such as ginseng with rusty root syndrome (Wei et al. 2020). Chytridiomycota enhances crop resistance, while Mucoromycota is linked to reduced disease resistance. In this study, the relative abundances of Chytridiomycota in DMS (diseased mature stage), LMS (latently infected mature stage), and HMS (healthy mature stage) were 0.28%, 11.69%, and 2.11%, respectively, while Mucoromycota abundances were 42.45%, 23.60%, and 30.74%.

The fungal species *Rhizopus arrhizus* and *Rhizopus microsporus* were significantly enriched in diseased mature plants. *Rhizopus arrhizus* is a known pathogen of sunflower rot (Abeywickrama et al. 2020) and postharvest bulb rot (Gao et al. 2024), while *R. microsporus* induces water-soaked, puffy, and dark-gray lesions in leaf mustard (Wang et al. 2020a, b). *Rhizopus microsporus* releases toxins that kill host cells, facilitating necrotrophic growth for both the fungus and associated bacteria (Partida-Martinez & Hertweck 2005). These observations suggest that *R. arrhizus* and *R. microsporus* may promote the proliferation of konjac soft rot pathogens, exacerbating disease severity.

### Ecological implications of microbial-soil factor correlations

The observed correlations provide mechanistic insights into microbial community assembly and functional adaptation in the konjac rhizosphere. Alphaproteobacterial taxa demonstrated alkaliphilic preferences potentially thriving in root exudate-induced alkaline microzones. This contrasts with *Acidobacteria*, which showed positive associations with available nitrogen and potassium, reinforcing their role as nutrient-cycling plant growth-promoting bacteria (PGPB; Kielak et al. 2016). The negative correlation between Ktedonobacteraceae and OM could indicate resource competition with fast-growing decomposers or sensitivity to pathogen-induced OM degradation.

For fungi, *Rhizopus* spp. positive correlations with pH and nutrients may facilitate their pathogenicity by optimizing metabolic activity in specific edaphic contexts. The negative correlation between *Aspergillus novofumigatus* and OM suggests niche differentiation from bacteria in carbon cycling, while *Podospora anserina*’s pH association may reflect adaptive radiation in alkaline rhizospheres. These patterns underscore the importance of soil physicochemistry in shaping microbiome structure, with potential cascading effects on disease suppression or promotion.

### Enzymatic pathways in pathogen-microbiome interactions

The observed correlations between microbial taxa and enzyme-encoding genes provide mechanistic insights into how rhizosphere communities influence konjac soft rot development. The positive correlations between *Rhizopus* spp. and cell wall-degrading enzymes (EC 4.2.2.10/3.2.1.4) align with their necrotrophic lifestyle, where pectate lyase-mediated degradation of middle lamellae and cellulase-driven cellulose hydrolysis facilitate tissue maceration (Partida-Martinez & Hertweck. 2005). This enzymatic cascade likely synergizes with *Pectobacterium* spp., as suggested by co-occurrence patterns in diseased rhizospheres (He et al. 2022), forming a “pathogen-enzyme feedback loop” that exacerbates soft rot progression.

Conversely, bacterial taxa like *Xanthomonadaceae_bacterium* and *Rhodanobacteraceae_bacterium* negatively correlate with cellulase genes (EC 3.2.1.4), potentially inhibiting carbon substrate release to limit pathogen growth. Such functional roles align with their reported contributions to nutrient cycling in healthy rhizospheres (Wei et al. 2019) and support the hypothesis that these bacteria maintain soil suppressiveness by competing for resources or modulating enzyme activity (Mendes et al. 2011).

The dual correlation pattern of *Podospora anserina* (positive with EC 4.2.2.10, negative with other enzymes) highlights the metabolic complexity within fungal communities. This may reflect adaptive strategies during pathogen-host interactions, such as resource partitioning or stress responses, analogous to the functional plasticity observed in other soil fungi (Liao et al. 2021).

Collectively, these findings reveal that rhizosphere microbes influence disease severity through coordinated regulation of cell wall-degrading enzymes. Pathogenic fungi leverage enzymatic activity for virulence, while specific bacteria may counteract this process, underscoring the potential of targeting microbial-enzyme networks for microbiome-based biocontrol (Gu et al. 2022).

### Impact of the rhizosphere microbial functions

The metagenome represents the collective genomic content of environmental microorganisms. Compared to other omics approaches, metagenomics enables more efficient and accurate predictions of soil microbial functions. Enhanced microbial metabolic capacity improves plant nutrient absorption and compound synthesis, which are critical for rapid plant growth (Prabha et al. 2019). KEGG annotation identified enriched pathways in carbohydrate metabolism (17.6% relative abundance) and amino acid biosynthesis (2.76%), with significant downregulation of “Amino Sugar and Nucleotide Sugar Metabolism” in DMS (*p* < 0.01), which suggests impaired glycan degradation capacity during severe infection, potentially limiting energy supply for plant defense responses. These processes underscore the role of rhizosphere microorganisms in ensuring nutrient supply for plant growth (Wei et al. 2019).

### Limitations of the study

Notably, the study’s retrospective classification of early-stage soil samples based on final plant health status introduces potential biases. While this method enables correlation of microbiome patterns with disease outcomes, it does not definitively establish causality. Observed microbial differences between “diseased-associated” and “healthy-associated” early samples could reflect pre-existing microbiome configurations that predispose plants to infection, or post-inoculation microbial responses to pathogen presence. The former scenario supports the hypothesis that microbiome assembly drives disease resistance/susceptibility, whereas the latter suggests that pathogens alter microbiome structure. Distinguishing between these mechanisms requires longitudinal, prospective sampling with concurrent pathogen load measurements, which was beyond the scope of this study.

While the current analysis focuses on bacterial and fungal communities, viral sequences such as Heunggongvirae were detected but not functionally characterized. Although their relative abundance was low, the potential roles of bacteriophages or other viruses in influencing bacterial pathogens or microbiome dynamics were acknowledged, citing Berendsen et al. (2012). It was noted that further viral metagenomics or co-culture experiments are needed to explore their specific impacts, leaving this as an open question for future research.

Moreover, the correlations between microbial taxa and soil properties provide observational insights but do not establish direct causation. For example, the negative correlation between Ktedonobacteraceae and OM could stem from microbial consumption, reduced root exudation in diseased plants, or other indirect factors (Bardgett & van der Putten 2014). Controlled greenhouse experiments with manipulated soil parameters (e.g., OM content, pH) or synthetic microbial communities would be essential to validate mechanistic links.

### Future directions

To address these limitations and enhance the findings, future research should implement time-series sampling from planting to disease onset, integrating quantitative PCR tracking of *Pectobacterium* and *Rhizopus* pathogens to clarify whether microbial shifts precede pathogen proliferation or are secondary to infection (Berendsen et al. 2012). Future studies could use gnotobiotic systems such as axenic konjac seedlings inoculated with synthetic communities to test the causal roles of Chloroflexi, Acidobacteria, and Rhizopus species in disease suppression or promotion. For example, inoculating healthy plants with pure cultures of Chloroflexi to assess their ability to reduce disease incidence (Bulgarelli et al. 2012). Targeted viral metagenomics could be conducted to identify bacteriophages associated with *Pectobacterium* or beneficial bacteria, exploring how viral predation influences microbiome stability and disease outcomes (Srinivasiah et al. 2015). Factorial trials could be designed to decouple the effects of OM, pH, and nutrient availability on microbial communities, such as amending soils with specific organic amendments to test whether enhancing Chloroflexi/Acidobacteria abundances via soil chemistry reduces susceptibility (Mendes et al. 2011). Early-life microbiome signatures like lower Chloroflexi in DIS samples could be leveraged to develop predictive models for pre-symptomatic disease risk assessment, with machine learning approaches used to identify robust biomarker combinations (Lundberg et al. 2012). Probiotic formulations containing Chloroflexi or Acidobacteria strains, either alone or in combination with biocontrol fungi like Trichoderma, should be investigated to suppress *Rhizopus* and *Pectobacterium* in field settings (Raaijmakers et al. 2009).

## Conclusions

This study systematically characterized the spatiotemporal dynamics of rhizosphere microbiomes during konjac soft rot progression using metagenomic approaches, revealing distinct microbial assembly patterns linked to disease outcomes. Key findings indicate that rhizosphere microbial communities exhibit significant divergence in taxonomic composition and functional profiles across plant health statuses, with diseased plants at maturity showing elevated microbial richness but reduced community evenness compared to healthy/latently infected counterparts.

At the phylum level, Chloroflexi and Acidobacteria were identified as putative biocontrol agents, with their relative abundances negatively correlated with disease severity. Conversely, Mucoromycota, particularly pathogenic *Rhizopus arrhizus* and *Rhizopus microsporus*, were enriched in diseased rhizospheres, suggesting their role in promoting tissue maceration. Functional annotation revealed that carbohydrate metabolism and amino acid biosynthesis pathways were consistently active across health states, underscoring the fundamental role of rhizosphere microbiomes in plant nutrient cycling.

This work provides a mechanistic foundation for developing microbiome-based disease management strategies, such as inoculation with Chloroflexi/Acidobacteria-enriched consortia or targeted suppression of *Rhizopus* spp. Future studies integrating gnotobiotic systems and longitudinal sampling will be critical to validate causal relationships between microbial keystone species and disease suppression, ultimately facilitating the transition toward sustainable, agrochemical-reduced konjac production.

## Supplementary Information

Below is the link to the electronic supplementary material.

Supplementary file1 (DOCX 21 KB)

## Data Availability

No datasets were generated or analysed during the current study.
