# Supplementary material for: Metagenomic analysis of microbial community dynamics in konjac rhizosphere during soft rot disease progression
Source: Appl Microbiol Biotechnol. 2025 Oct 3;109(1):212. doi: 10.1007/s00253-025-13600-4 (PMC12494664; doi:10.1007/s00253-025-13600-4)
Supplement: Supplementary file 1 — Supplementary file1 (DOCX 21 KB) [file 253_2025_13600_MOESM1_ESM.docx]

**Supplementary information**

**Supplementary table 1 The soil physico-chemical properties in the rhizosphere of konjac under different health statuses and growth stages.**

| Sample | Available phosphorus mg/kg | Available nitrogen mg/kg | Available potassium mg/kg | Organic matter % | pH value |
| --- | --- | --- | --- | --- | --- |
| HIS | 42.35 ± 4.96 d | 145.83 ± 10.69 b | 105.14 ± 5.00 b | 2.64 ± 0.11 d | 4.03 ± 0.03 e |
| HVS | 59.54 ± 4.23 cd | 111.42 ± 2.67 c | 92.41 ± 2.00 c | 2.89 ± 0.03 c | 4.18 ± 0.02 c |
| HMS | 136.43 ± 2.88 b | 79.92 ± 14.25 d | 59.75 ± 1.00 f | 3.26 ± 0.03 ab | 4.12 ± 0.02 d |
| DIS | 66.22 ± 9.22 c | 172.08 ± 6.15 a | 115.07 ± 5.00 a | 3.26 ± 0.09 ab | 4.54 ± 0.04 a |
| DVS | 41.84 ± 4.86 d | 109.08 ± 4.40 c | 84.61 ± 4.00 d | 3.11 ± 0.05 b | 4.09 ± 0.01 d |
| DMS | 74.74 ± 6.12 c | 77.58 ± 3.64 d | 67.99 ± 3.00 e | 3.32 ± 0.03 a | 4.26 ± 0.01 b |
| LIS | 14.37 ± 4.59 e | 109.67 ± 10.10 c | 83.27 ± 3.00 d | 2.15 ± 0.03 e | 4.09 ± 0.01 d |
| LVS | 60.35 ± 3.82 cd | 78.17 ± 5.35 d | 85.04 ± 5.00 d | 2.54 ± 0.26 d | 4.11 ± 0.01 d |
| LMS | 350.79 ± 28.07 a | 70.58 ± 8.98 d | 43.98 ± 3.00 g | 2.32 ± 0.11 e | 4.26 ± 0.01 b |
